# Preservation of proliferating pancreatic progenitor cells by Delta-Notch signaling in the embryonic chicken pancreas

**DOI:** 10.1186/1471-213X-7-63

**Published:** 2007-06-07

**Authors:** Jonas Ahnfelt-Rønne, Jacob Hald, Anne Bødker, Hani Yassin, Palle Serup, Jacob Hecksher-Sørensen

**Affiliations:** 1 Department of Developmental Biology, Hagedorn Research Institute, Niels Steensensvej 6, DK-2820 Gentofte, Denmark

## Abstract

**Background:**

Genetic studies have shown that formation of pancreatic endocrine cells in mice is dependent on the cell autonomous action of the bHLH transcription factor Neurogenin3 and that the extent and timing of endocrine differentiation is controlled by Notch signaling. To further understand the mechanism by which Notch exerts this function, we have investigated pancreatic endocrine development in chicken embryos.

**Results:**

In situ hybridization showed that expression of Notch signaling components and pro-endocrine bHLH factors is conserved to a large degree between chicken and mouse. Cell autonomous inhibition of Notch signal reception results in significantly increased endocrine differentiation demonstrating that these early progenitors are prevented from differentiating by ongoing Notch signaling. Conversely, activated Notch1 induces *Hes5-1 *expression and prevents endocrine development. Notably, activated Notch also prevents Ngn3-mediated induction of a number of downstream targets including *NeuroD*, *Hes6-1*, and *MyT1 *suggesting that Notch may act to inhibit both *Ngn3 *gene expression and protein function. Activated Notch1 could also block endocrine development and gene expression induced by NeuroD. Nevertheless, Ngn3- and NeuroD-induced delamination of endodermal cells was insensitive to activated Notch under these conditions. Finally, we show that Myt1 can partially overcome the repressive effect of activated Notch on endocrine gene expression.

**Conclusion:**

We conclude that pancreatic endocrine development in the chicken relies on a conserved bHLH cascade under inhibitory control of Notch signaling. This lays the ground for further studies that take advantage of the ease at which chicken embryos can be manipulated.

Our results also demonstrate that Notch can repress Ngn3 and NeuroD protein function and stimulate progenitor proliferation. To determine whether Notch in fact does act in Ngn3-expressing cells *in vivo *will require further studies relying on conditional mutagenesis.

Lastly, our results demonstrate that expression of differentiation markers can be uncoupled from the process of delamination of differentiating cells from the epithelium.

## Background

The pancreas is an organ containing both exocrine and endocrine cell populations. The exocrine pancreas consists of acini and ducts that produce and transport enzymes and bicarbonate to the digestive tract. Lineage tracing studies have revealed that both endocrine and exocrine cells are derived from *Pdx1*-expressing progenitors [1-3]. The endocrine cells are organized in the islets of Langerhans which contain five distinct cell types, each characterized by the production of specific peptide hormones [4-6]. Endocrine cells begin to appear soon after the first morphological signs of pancreas formation which occurs approximately at the 25-somite stage in the mouse and chicken [7]. Endocrine development depends on *Neurogenin3 *(*Ngn3*) and is initiated by the onset of *Ngn3 *expression in a subset of pancreatic progenitor cells [8-11]. All endocrine cells are derived from *Ngn3 *expressing precursors and the vast majority, if not all, of the *Ngn3 *expressing cells are committed to the endocrine lineage [1,12]

Several studies have demonstrated that Notch signaling is involved in the development of endocrine cells in the pancreas. Mice harboring mutations in the Notch pathway genes *Dll1*, *RBK-Jκ*, and *Hes1 *all display precocious and excessive endocrine development as early as E9.5 [10,13]. At this stage there is an increase in the numbers of *Ngn3 *positive cells in *Dll1 *and *RBP-Jκ *mutants, and at E10.5 an increase in endocrine cells. *Ngn3 *expression was not analyzed in *Hes1 *mutants but these had increased numbers of glucagon-producing cells at E9.5. Together these studies suggest that Notch signaling prevents endocrine differentiation through a mechanism known as lateral inhibition where the Notch ligand Dll1, expressed in differentiating cells, signals through Notch receptors on adjacent cells thereby keeping them undifferentiated or acquiring a secondary fate. However, Notch signaling may act to regulate differentiation by controlling the proneural genes at the transcriptional level or Notch signaling could act in already committed precursors by inhibiting proneural factors posttranslationally. These are not mutually exclusive mechanisms and indeed both have been proposed [14-16]. Later studies have suggested that Notch signaling not only regulates endocrine specification but also inhibits exocrine differentiation [17,18]. This was further supported by the finding that *Hes1*, a Notch target gene, is active in exocrine precursors in the mouse pancreas and prevents their terminal differentiation, and that loss of Notch signaling in zebrafish accelerate exocrine differentiation [19]. Subsequently, Jagged mediated Notch signaling has been suggested to mediate a fate choice between exocrine and intrapancreatic duct fate from a common precursor cell in zebrafish [20]. This is most likely different from the situation in mice where duct cell progenitors and exocrine progenitors appear to diverge very early in development between E9.5 and E11.5, prior to expression of Jagged-1 [1,21].

Here we show that the expression of pro-endocrine bHLH factors and Notch pathway components is conserved in the embryonic chicken pancreas and identify *Hes6-1 *as being expressed in the endocrine lineage in the embryonic pancreas. We demonstrate that inhibition of Notch signaling results in increased endocrine differentiation, and that activated Notch1 (Notch1^ICD^) blocks endocrine development and maintains proliferation of pancreatic progenitor cells in the embryonic chicken pancreas. We demonstrate that Notch1^ICD ^is able to inhibit Ngn3 activity as it prevents Ngn3 induced endocrine differentiation, visualized by loss of *NeuroD*, *Myt1*, *Hes6-1*, Pax6, βIII-tubulin, and glucagon expression but, remarkably, without affecting delamination of the Ngn3-expressing cells from the gut epithelium. We also show that NeuroD-induced endocrine development is sensitive to inhibition by Notch. Lastly, we show that forced expression of *Myt1 *partly restores βIII-tubulin expression in Notch1^ICD ^expressing cells. Together, our results demonstrate that Notch regulation of pancreatic endocrine development is conserved in chicken, and raises a possibility of Notch mediated inhibition of Ngn3 and NeuroD protein activity.

## Results

### The embryonic chicken pancreas expresses pro-endocrine bHLH genes

**Figure 1:**
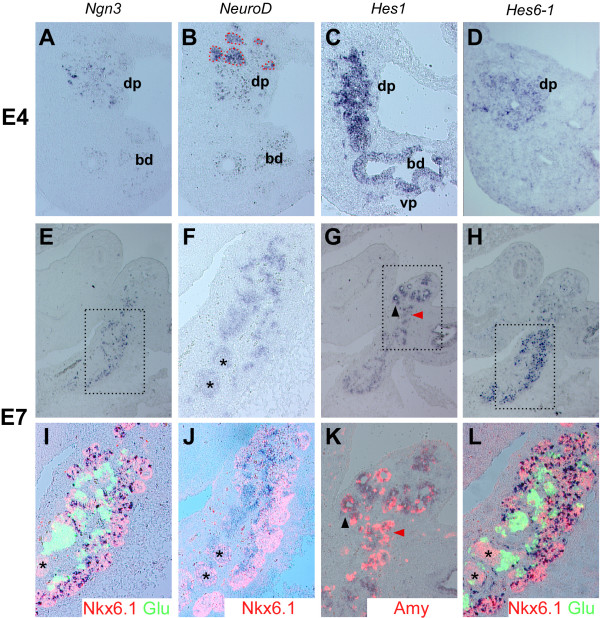
**Expression of bHLH genes in the chicken pancreas**. (A-L) In situ hybridization on serial sections from HH st. 22 (A-D) and HH st. 31 (E-L) chick pancreas showing the expression patterns of *Ngn3 *(A, E, I), *NeuroD *(B, F; J), *Hes1 *(C, G, K), *Hes6-1 *(D, H, L). Dorsal (dp) and ventral pancreas (vp) and the bile duct (bd) are indicated in A-D. At HH st. 22, *Ngn3 *is expressed in a few cells scattered in the epithelium (A) whereas *NeuroD *expression is confined to endocrine cell clusters located at the most distal part of the dorsal pancreas (red dashed lines in B). *Hes1 *is expressed broadly in the dp and vp epithelium as well as in the bd epithelium (C). *Hes6-1 *is expressed in a pattern resembling *Ngn3 *(compare A to D). At HH st. 31, *Ngn3 *is expressed in scattered cells within the epithelium (E) where they co-localize with Nkx6.1 (I). *Ngn3 *is not expressed in glucagon positive cells adjacent to the Nkx6.1 positive epithelium (I). In contrast, *NeuroD *expressing cells are confined to endocrine cells and are excluded from the Nkx6.1 positive epithelium (F, J, area corresponding to boxed areas in E, H). *Hairy-1 *is still expressed broadly in the epithelium (G) and some of the forming amylase positive acini are also positive for *Hes1 *(black arrowhead in G, K) but amylase positive acini without *Hes1 *expression can also be found (red arrowhead in G, K). *Hes6-1 *is expressed in a pattern closely resembling *Ngn3 *(H) as it co-localizes with Nkx6.1 in the epithelium and is excluded from glucagon positive endocrine cells (L). * denotes Nkx6.1 positive β-cells

Pancreatic endocrine development in the mouse relies on the pro-endocrine genes *Ngn3 *and *NeuroD *[1,8-10,22] whose activity is, at least partly, controlled by Hes1 and Notch signaling [10,13,17,18]. To determine if these genes are expressed in the chicken pancreas we performed in situ hybridizations on sections of HH st. 22 [23] (E4) and HH st. 31 (E7) chicken pancreas (Fig. 1). These timepoints correspond roughly to E11 and E14 of mouse pancreas development where the epithelium expands and the endocrine and exocrine differentiation peaks, respectively. At HH st. 22, *Ngn3 *was expressed in a few scattered cells within the epithelium (Fig. 1A) while *NeuroD *was expressed in clusters of endocrine cells located at the distal part of the dorsal pancreatic bud (Fig. 1B) as determined by co-staining for Nkx6.1, a marker of pancreatic progenitor cells as well as β-cells, and glucagon expressed by the α-cells (not shown). The chicken *Hes1 *homologue, formerly known as *Hairy1 *[24], now *Hes1 *after official nomenclature, was expressed broadly in the pancreatic epithelium at HH st. 22 (Fig. 1C) while *Hes6-1 *was expressed in scattered cells within the epithelium (Fig. 1D). In the HH st. 31 pancreas, the overall expression patterns were similar to the HH st. 22 pancreas (Fig. 1E–L), but we consistently observed that *Hes1 *expression levels varied between different regions of the epithelium such that areas with low levels of *Hes1 *expression corresponded to regions where *Ngn3 *and *Hes6-1 *were expressed (Fig. 1E,G,H). To further define the cell types that expressed the four bHLH genes we used immunohistochemical detection of glucagon, amylase, and Nkx6.1 on sections previously subjected to in situ hybridization. We found that regions expressing high levels of *Hes1 *(boxed area in fig. 1G) were devoid of glucagon (data not shown), whereas amylase was readily detectable in *Hes1*-expressing cells (Fig. 1K). *Ngn3 *and *Hes6-1 *were both expressed in pancreatic progenitor cells marked by Nkx6.1 [25,26], but absent from glucagon-producing cells (Fig. 1I and 1L). *NeuroD *expression was restricted to clusters of endocrine cells (including Nkx6.1^+ ^insulin-producing cells) adjacent to the Nkx6.1 positive epithelium (Fig. 1F,J). We also analyzed the expression of Hes6-2 and Hes5-1, -2, and -3 [27], but could not detect any transcripts in the pancreas. Lastly, using whole-mount in situ hybridization on micro-dissected HH st. 22 chicken pancreas we observed expression of *Delta1*, *Notch1*, and the chicken *Jagged-1 *and *-2 *homologs *Serrate-1 *and *-2 *(data not shown), but due to technical limitations we were not able to further define the cell types that expressed these genes.

### Forced expression of Notch ligands induces endocrine differentiation

**Figure 2:**
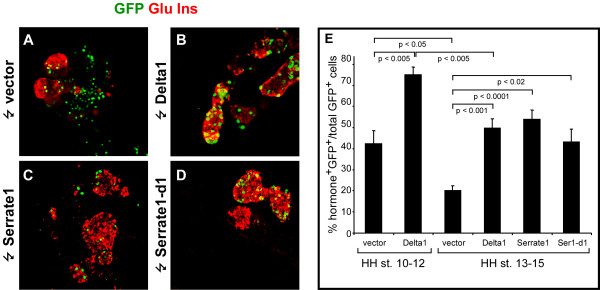
**Inhibition of Notch signaling induce excess endocrinedifferentiation**. (A-D). Confocal sections of chicken embryonic pancreata 72 hours after electroporation with control vector (A), Delta1 (B), Serrate1 (C), and Serrate1-d1 (D) subjected to immunohistochemical stainings against GFP (green) and glucagon plus insulin (red). Cells expressing GFP from the vector readily co-localize with endocrine markers. However, most electroporated cells are not endocrine (A). In contrast, electroporation with either Delta1, Serrate1, or Serrate-d1 results in increased endocrine differentiation of electroporated cells as compared to the control (compare B, C, D to A). E. Quantitative analysis of endocrine differentiation 72 hours after electroporation with the indicated expression plasmids at HH st. 10–12 or HH st. 13–15. (F, G).

**Figure 3:**
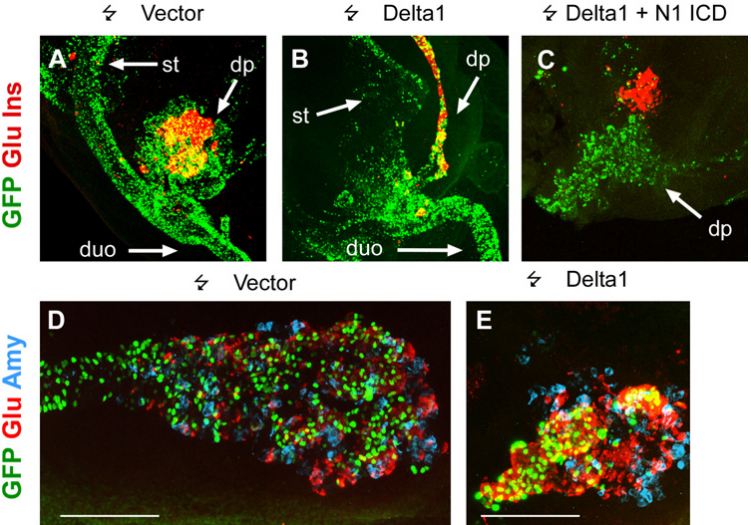
**Cell autonomous effects of ligand expression is reversed by Notch1^ICD^**. 3D projections of confocal optical sections of chicken embryos subjected to whole mount immunohistochemical stainings for GFP (green) and glucagon plus insulin (red) (A-C) or GFP (green), glucagon (red) and amylase (blue) (D, E) 72 hours after electroporation with the indicated constructs. The most extreme phenotype resulting from ectopic Delta1 expression is an almost complete conversion of the pancreas into a tubular structure composed almost entirely of endocrine cells (compare B to A). When Delta1 is co-expressed with Notch1^ICD ^most transfected cells do not express the hormones (C). D and E show the entire dorsal pancreas at the same magnification (scale bar represents 100 μm). Note the even distribution of electroporated cells in the control (D) and the clustering of electroporated cells in the endocrine population in the Delta1 electroporated pancreas.

Expression of pro-endocrine and Notch pathway genes suggests that Notch signaling might regulate endocrine development in the chicken pancreas. If the function of Delta1 is to signal to adjacent progenitor cells and prevent them from entering the endocrine differentiation program, then forced widespread expression of Delta1 in the pancreatic epithelium should reduce the number of endocrine cells formed. To test this prediction, we introduced plasmids expressing Delta1 upstream of an IRES-GFP cassette into the endoderm of HH st. 10–12 and HH st. 13–15 embryos by *in ovo *endoderm electroporation [11] and assayed pancreatic endocrine differentiation after 72 hours (at HH st. 26–27) by staining for GFP, insulin, and glucagon (Fig. 2). Embryos electroporated with an IRES-GFP vector lacking the insert served as controls. We found that extensive expression of Delta1 in both HH st. 10–12 and HH st. 13–15 embryos resulted in a marked increase in the fraction of GFP-expressing cells that differentiate into endocrine cells, compared to controls (Fig. 2A,B and 2E). At a first glance this result may seem paradoxical, but we speculate that a cell autonomous dominant negative action of high levels of Delta1 on Notch signaling [28-33] results in increased endocrine differentiation. Support for such a cell autonomous dominant negative effect comes from additional experiments where we introduced either Serrate1, or a C-terminally truncated form of Serrate1, (Serrate1-d1), lacking signaling activity and previously shown to act in a cell autonomous dominant negative fashion [28]. Such embryos showed an increase in endocrine differentiation that was comparable to that seen with Delta1 regardless of whether full-length or truncated Serrate1 was used (Fig. 2, compare C, D to A, B), arguing that the differentiation inducing effects seen when over-expressing full-length Delta1 and Serrate1 is the result of a cell autonomous inhibition of Notch signal reception. Similar experiments using a truncated Delta1 construct (Delta1-d1, [28]) were inconclusive as very few GFP^+ ^cells remained 48 hours after electroporation and none were detectable after 72 hours. Quantitatively, mis-expression of Delta1 beginning at HH st. 10–12 or HH st. 13–15 resulted in an increase of the fraction of endocrine GFP^+ ^cells from ~40% to ~75% and from ~20% to ~50%, respectively (Fig. 2E). In extreme cases pancreas morphology was severely altered with the dorsal pancreas adopting a tubular morphology with the epithelium composed almost exclusively of endocrine cells (Fig. 3 compare B to A). Notably, forced expression of Delta1 had no endocrinogenic effect in the prospective duodenum (Fig. 3B). If the effect of the ligands were a cell-autonomous inhibition of Notch signaling then this effect should be blocked by a constitutive activation of the Notch pathway in the same cell. To test this, we co-expressed Delta1 with a constitutively active form of Notch1 (Notch1^ICD^)[17] in the chick pancreas by *in ovo *electroporation. In agreement with this model we found that Notch1^ICD ^was able to reverse the effect of Delta on endocrine differentiation such that most transfected cells were excluded from the endocrine area under these conditions (Fig. 3B,C).

To test if ectopic expression of the ligands also induced exocrine differentiation we compared the expression of the exo- and endocrine markers amylase and glucagon, respectively, in control and Delta1 electroporated chicken pancreata (Fig 3D,E). GFP-expressing cells were evenly distributed throughout the pancreas epithelium, including exocrine and endocrine cells in the control pancreas. In contrast, the majority of the GFP positive cells expressed glucagon and were excluded from the exocrine cells in Delta electroporated pancreata. Furthermore, the Delta1-expressing pancreata were consistently smaller, suggesting that Delta1 induces precocious endocrine differentiation at the expense of progenitor expansion and later born cell types such as the exocrine.

### Constitutively active Notch1 prevents endocrine differentiation and maintains proliferation

**Figure 4:**
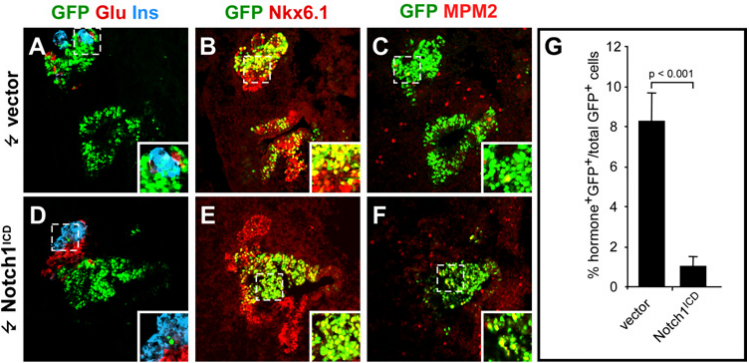
**Notch1^ICD ^directly regulates proliferation and blocks endocrine differentiation**. (A-F). Confocal sections of chicken embryonic pancreata 52 hours after electroporation with empty vector (A-C) or Notch1^ICD ^(D-F) subjected to immunohistochemical stainings against glucagon (red), and insulin (blue) (A and D), Nkx6.1 (red) (B and E), and MPM2 (red) (C and F). GFP was detected by its endogenous fluorescence (A-F). Inserts in A-F show higher magnifications of the boxed areas in the cognate panels. (G) Bar graph illustrating the quantitative analysis of endocrine differentiation 52 hours after electroporation with control vector or Notch1^ICD ^plasmid at HH st. 13–15. Note the absence of insulin and glucagon and the presense of MPM2 in GFP-positive cells in the Notch1^ICD ^electroporated embryos.

The results above suggest that Notch signaling inhibits endocrine differentiation in the chicken pancreas and that this inhibitory signal must be terminated before differentiation can occur. To test the effect of a constitutive activation of Notch signaling on normal pancreas development we introduced Notch1^ICD ^[17] by *in ovo *endoderm electroporation at HH st. 13–15 and analyzed endocrine development after 52 hours (HH st. 21–23)(Fig. 4). We found that GFP^+ ^cells in embryos expressing the control vector readily contributed to α- and β-cells as determined by co-localization of GFP with glucagon and insulin (Fig. 4A), while GFP^+ ^cells in Notch1^ICD^-expressing embryos were only rarely found to co-express glucagon or insulin (Fig. 4D). Quantitative analysis revealed that only ~1% of the GFP^+ ^cells in the Notch1^ICD^-expressing pancreas were endocrine compared to 8% for control embryos (Fig. 4G), demonstrating that Notch1^ICD ^blocks endocrine differentiation also in the chicken pancreas. The few cells we did find co-expressing Notch1^ICD ^and endocrine markers can be explained by the occasional electroporation of existing endocrine cells at the time of electroporation [11].

**Figure 5:**
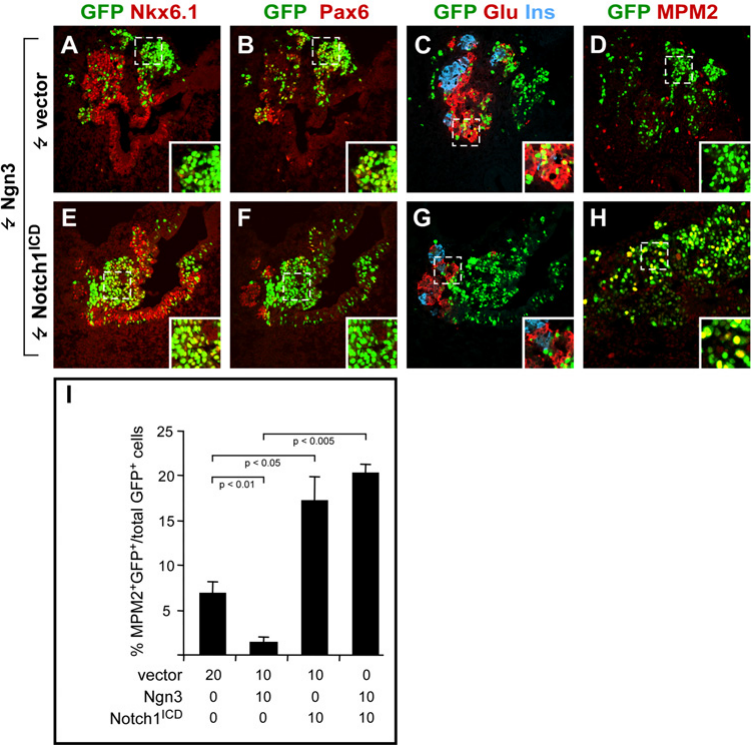
**Notch1^ICD ^inhibits the activity of Ngn3**. (A-H). Confocal sections of chicken embryonic pancreata 52 hours after electroporation with Ngn3 (A-D) or Ngn3 and Notch1^ICD ^(E-H) subjected to immunohistochemical stainings against Nkx6.1 (red) (A, E), Pax6 (red) (B, F), glucagon (red) and insulin (blue) (C, G), or MPM2 (red) (D, H). GFP was detected by its endogenous fluorescence (A-H). Inserts show higher magnification of boxed areas in the cognate panels. (I) Bar graph illustrating the quantitative analysis of MPM2/GFP double-positive cells as a percentage of total GFP-positive cells 52 hours after electroporation with the indicated expression plasmids at HH st. 13–15. Note the absence of insulin and glucagon, the presense of MPM2, and persistent Nkx6.1 expression in GFP-positive cells in the Ngn3 plus Notch1^ICD ^electroporated, compared to Ngn3 electroporated embryos.

In addition to reduced endocrine development we also observed that the morphology of the pancreas anlage in Notch1^ICD^-expressing embryos was disturbed compared to control embryos, such that the Nkx6.1^+ ^pancreas epithelium appeared expanded and highly folded compared to controls (Fig. 4, compare B and E). We hypothesized that the folded epithelium we observed in Notch1^ICD ^expressing pancreas could be the result of increased proliferation. We therefore analyzed proliferation of the Nkx6.1^+ ^pancreas epithelium using a monoclonal antibody against MPM2 which is a marker for mitotic cells [34]. We found that the fraction of Notch1^ICD^-expressing cells in the pancreas epithelium that expressed MPM2 was 2.5-fold higher than for corresponding cells expressing the control plasmid (Fig. 4C,F and Fig. 5I), demonstrating that active Notch signaling inhibits differentiation which is coincident with ongoing proliferation of pancreatic epithelial cells. These data lend further support to the notion of Notch signaling acting primarily to maintain a progenitor state, rather than inducing an alternative differentiated cell fate [35].

### Ngn3 induced cell cycle withdrawal and differentiation is antagonized by Notch1^ICD^

The results described above suggest that Notch mediated control of endocrine pancreas development is conserved in chicken. Previous results obtained in mice have indicated that Notch signaling acts to repress *Ngn3 *expression and thereby prevents endocrine differentiation [10,17,18]. However, it is also possible that Notch signaling may additionally act in cells that already have initiated *Ngn3 *expression by antagonizing the function of the encoded protein Ngn3 [16]. To test if activated Notch could interfere with the ability of Ngn3 to induce endocrine differentiation we took advantage of the ability of ectopic Ngn3 to induce endocrine differentiation in the chick endoderm [11]. We confirmed that Ngn3 induced endocrine differentiation in the chick pancreas (Fig. 5). Most Ngn3 positive cells were Nkx6.1 negative and Pax6 positive, suggesting they had differentiated into α-cells (Fig. 5A,B). In agreement with this we found that many Ngn3 positive cells expressed glucagon while insulin-expressing cells were rare (Fig. 5C). Mitotic cells were extremely rare among the Ngn3-expressing cells as judged by MPM2 immunoreactivity (Fig. 5D,I) similar to the situation in mouse where early endocrine cells are post mitotic [22]. To test if active Notch signaling can prevent Ngn3-induced endocrine differentiation in the chick pancreas, we co-electroporated HH st. 13–15 embryos with Ngn3 and Notch1^ICD ^expression vectors and assayed endocrine development after 52 hours. In contrast to embryos electroporated with the Ngn3 vector alone we found that most co-electroporated cells maintained Nkx6.1 expression (Fig. 5E) and displayed a high percentage of MPM2 expression (Fig. 5H,I) whereas very few cells expressed Pax6, glucagon or insulin (Fig. 5F,G). This result demonstrates that activated Notch1 can inhibit the function of the Ngn3 protein.

### *Hes5-1 *and *Hes6-1 *is inversely regulated by Ngn3 and Notch1^ICD^

**Figure 6:**
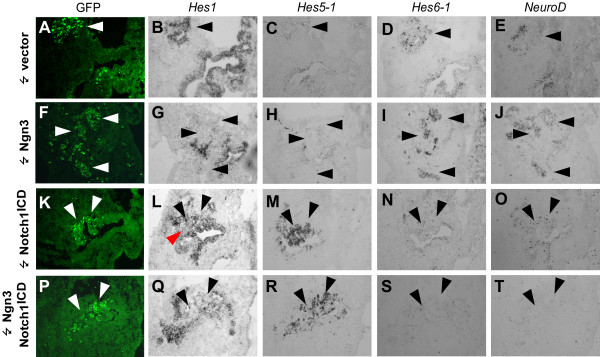
**bHLH genes are inversely regulated by Ngn3 and Notch1^ICD^**. (A-T). In situ hybridization on serial sections from embryonic pancreata 52 hours after electroporation with control vector (A-E), Ngn3 (F-J), Notch1^ICD ^(K-O), or Ngn3 and Notch1^ICD ^(P-T). The expression patterns of *Hes1 *(B, G, L, Q)), *Hes5-1 *(C, H, M, R), *Hes6-1 *(D, I, N, S), and *NeuroD *(E, J, O, T) were examined. A, F, K, P show GFP expressing electroporated cells (arrowheads) and the corresponding area is indicated with arrowheads in the rest of the panels. Note that Notch1^ICD ^apparently leads to decreased *Hes1 *expression (red arrowhead in L and arrowheads in Q) and induces *Hes5-1 *expression (M, R) and repress *Hes6-1 *(N, S) and *NeuroD *(O, T) expression regardless of the presence of Ngn3.

To begin to unravel the mechanism behind Notch-mediated inhibition of endocrine development, we next investigated the expression pattern of *Hes1*, *Hes5-1*, *Hes6-1*, and *NeuroD *in the pancreas after introduction of Ngn3, Notch1^ICD ^and Ngn3 + Notch1^ICD ^or control vector (Fig. 6). The expression pattern of these genes was not affected in embryos electroporated with control vector (compare Fig. 6B,D,E to Fig. 1B,C,D and not shown for *Hes5-1*). Conversely, Ngn3 electroporation led to loss of *Hes1 *expression and a robust induction of *NeuroD *and *Hes6-1 *in the electroporated area (Fig. 6G,I,J). In contrast, Notch1^ICD ^repressed *NeuroD *and *Hes6-1 *expression in the electroporated area (Fig. 6N,O). Notably, we found no evidence to suggest that Notch1^ICD ^up-regulated *Hes1 *(Fig. 6L) and it even appeared to be down-regulated in the transfected area. Instead, *Hes5-1*, another Notch target gene [27,36] not normally expressed in the pancreas epithelium, was strongly induced in Notch1^ICD ^expressing cells (Fig. 6M). In the developing nervous system of the mouse, *Hes1 *and *Hes5 *are expressed in complementary patterns and *Hes5 *expression is expanded in the Hes1 null mice and vice versa indicating mutual cross repression [37]. *Hes5 *expression is also induced in the endoderm of Hes1 null mice [13]. In the chick embryo *Hes5-*1 is known to repress the expression of other chicken Hes genes [27] and it is possible that it also respresses *Hes1 *under these conditions. Similar results were recently reported by Matsuda et al. who found that ectopic expression of Notch1^ICD ^in chick embryonic stomach endoderm failed to up-regulate transcription of *Hes1 *and *Hairy2*, two Hes homologues normally found to be expressed in this tissue [38]. When Ngn3 and Notch1^ICD ^were co-electroporated we observed a robust induction of *Hes5-1 *expression while Ngn3-induced *NeuroD *and *Hes6-1 *expression was lost (Fig. 6Q–T). *Hes1 *also appeared down-regulated under these conditions.

### Activated Notch1 inhibits Ngn3 and NeuroD induced endocrine differentiation but not delamination

**Figure 7:**
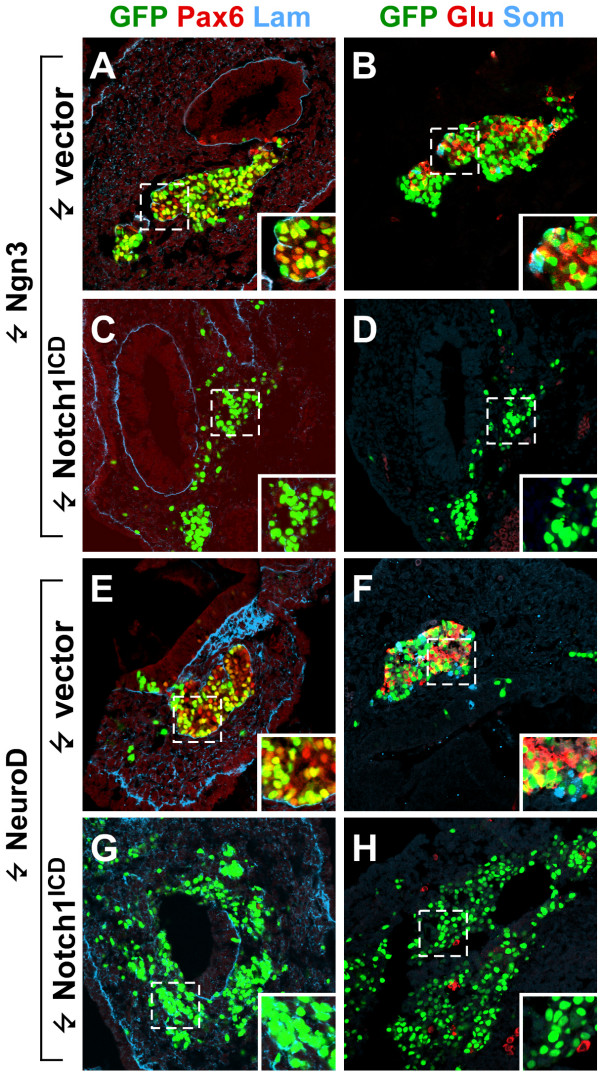
**Differentiation can be uncoupled from delamination**. (A-H). Confocal sections of chicken embryonic pancreata 52 hours after electroporation with Ngn3 (A-B), Ngn3 and Notch1^ICD ^(C-D), NeuroD (E-F), or NeuroD and Notch1^ICD ^(G-H) subjected to immunohistochemical stainings against Pax6 (red) and laminin (blue) (A, C, E, G) and glucagon (red) and somatostatin (blue) (B, D, F, H). GFP was detected by its endogenous fluorescence (A-H). Inserts show higher magnification of boxed areas in the cognate panels. Note that Notch1^ICD ^inhibits Ngn3 and NeuroD induced expression of endocrine markers without affecting delamination of the cells from the endoderm.

Our finding that Notch1^ICD ^inhibits both expression and function of Ngn3 is consistent with previous reports showing that X-Notch^ICD ^inhibits both the expression and function of the Ngn3 related X-NGNR-1 during *Xenopus *neurogenesis [39,40]. Furthermore, since X-NGNR-1 can induce *XNeuroD *expression while the converse is not the case [39] it appears that a unidirectional bHLH cascade regulates neural determination and differentiation (regulated by Neurogenin and NeuroD, respectively). Whether either or both of these processes are repressed by Notch signaling is not fully understood. Furthermore, it is still unknown whether NeuroD can induce *Ngn3 *expression, and the sensitivity of these bHLH factors to Notch mediated inhibition in endocrine development has not previously been investigated. To test if the Neurogenin-NeuroD cascade is unidirectional in endocrine development we compared the effect of introducing Ngn3 and NeuroD. We analyzed *Ngn3 *expression by in situ hybridization in NeuroD electroporated cells but found no evidence to suggest that NeuroD induce *Ngn3 *expression (not shown), consistent with similar studies in the nervous system [39] and indicating that NeuroD activates the differentiation program independent of *Ngn3*. However, we cannot exclude a transient induction of *Ngn3 *at earlier timepoints not analyzed. Next we examined if Notch1^ICD ^could block the differentiation induced by Ngn3 or NeuroD by performing co-electroporation experiments (Fig. 7). In these experiments we targeted the prospective duodenum where no endocrine development occurs at the stages examined unless Ngn3 is induced and where delamination of differentiating endocrine cells is more readily scored [11]. We used an antibody to laminin to visualize the basal lamina of the endodermal epithelium and Pax6, glucagon, somatostatin and neuronal classIII β-tubulin [41] specific antibodies as endocrine markers. In our experiments both Ngn3 and NeuroD induced endocrine differentiation as evidenced by Pax6-, glucagon-, somatostatin-, and neuronal classIII β-tubulin expression and delamination of the differentiated cells (Fig. 7A,B and 7E,F). However, when Notch1^ICD ^was co-expressed with either Ngn3 or NeuroD we found an almost complete loss of all these endocrine markers (Fig. 7C,D and 7G,H), showing that during forced endocrine differentiation both Ngn3 and NeuroD function is sensitive to Notch. Delamination of Ngn3 and NeuroD expressing cells still occurred under these conditions (Fig. 7, compare A to C and E to G), showing that delamination and expression of differentiation markers can be uncoupled during endocrine development.

### Myt1 can partially antagonize the effects of Notch1^ICD ^on Ngn3 function

**Figure 8:**
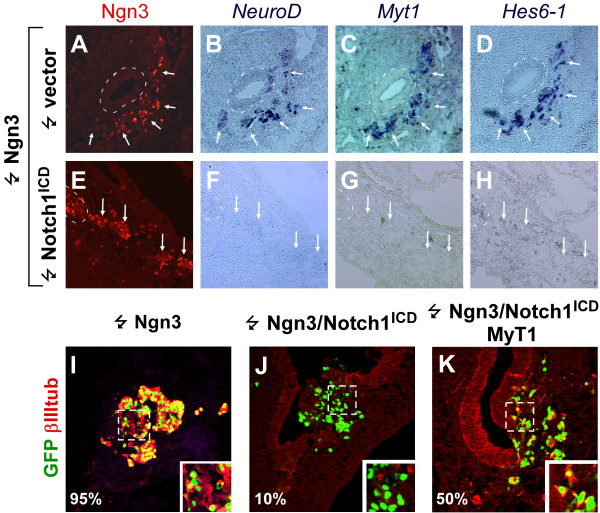
**Myt1 can partially rescue the inhibitory effect by Notch1^ICD ^on Ngn3 function**. (A-K). Immunohistochemical stainings (A, E, I-K) and in situ hybridizations (B-D, F-H) on sections in the duodenum 52 hours after electroporation with Ngn3 (A-D, I), Ngn3 and Notch1^ICD ^(E-H, J) or Ngn3 with Notch1^ICD ^and MyT1 (K) showing expression of Ngn3 (A, E), *NeuroD *(B, F), *Myt1 *(C, G), *Hes6-1 *(D, H), and βIII-tubulin. Note that Ngn3 fails to induce expression of endocrine markers in the presence of Notch1ICD. βIII-tubulin expression is partially restored when Notch1^ICD ^and MyT1 are co-expressed with Ngn3 (compare K to J and I).

The above experiments demonstrate that activated Notch1 can inhibit both Ngn3- and NeuroD-induced endocrine development. Since certain Ngn3-induced gene products, such as Hes6-1 and MyT1, have been reported to attenuate Notch mediated inhibition [42,43] we wanted to analyze if expression of any of these genes was insensitive to Notch inhibition in the duodenum, thus possibly explaining the delamination observed in the presence of activated Notch. As mentioned above (Fig. 6 and 7), both native and Ngn3-induced *NeuroD *and *Hes6-1 *expression in the pancreas is sensitive to Notch inhibition but delamination is not readily scored in the pancreas due to the branching of the epithelium. We therefore repeated the experiment in the duodenum and found that Ngn3-induced expression of these two genes as well as *MyT1 *was repressed by Notch1^ICD ^also at that location (Fig. 8A–H). This result suggests that Ngn3 can initiate a delamination program independent of the activity of these genes and that Ngn3 may have actions insensitive to Notch repression.

The levels of Notch1^ICD ^resulting from forced expression are likely much higher than found during native Notch signaling events. Our experiments therefore do not rule out that Hes6-1 and/or MyT1 could render cells refractive to the action of more physiological levels of Notch as seen in neurogenesis [43]. To test if forced expression of MyT1 at high levels could antagonize Notch1^ICD ^function we electroporated chicken endoderm with constructs encoding Ngn3, Notch1^ICD ^and a FLAG tagged Nzf2b isoform of Myt1. This is one of two Myt1 isoforms resulting from alternative transcriptional start sites reported to be expressed at highest levels in the mouse pancreas. Furthermore, it is the only isoform capable of initiating the endocrine differentiation program in the chicken endoderm [44]. We found that expression of MyT1 led to a partial restoration of neuronal classIII β-tubulin expression (Fig. 8I–K), indicating that some aspects of endocrine differentiation have been rescued in these cells. Expression of Pax6, glucagon and somatostatin was, however, not restored under these conditions (not shown), suggesting that other factors are needed to overcome the repression by Notch1^ICD^.

## Discussion

The pancreatic expression of bHLH genes and their sensitivity to Notch mediated inhibition observed in this study suggests that endocrine development is highly conserved between mice and chicken.

### Overexpression of Notch ligands leads to endocrine differentiation

The observed stimulatory effect of Delta1 over-expression upon endocrine differentiation is in contrast to the effect of Delta1 over-expression in the chicken retina where widespread RCAS-mediated expression of Delta1 inhibits neuronal differentiation [45], and seemingly at odds with the proposed function of Delta1 as a signal that inhibits endocrine differentiation. However, high level expression of Notch ligands has previously been reported by a number of groups to result in cell autonomous inhibition of Notch signal reception [28-33], raising the possibility that the effect we observe is cell autonomous and the result of inhibition of Notch signaling rather than stimulation of Notch signaling in neighboring cells. In our experiments we used plasmid-based electroporation which results in multiple copies being taken up by the electroporated cells which combined with the very strong promoter used may result in expression levels exceeding those obtained by Henrique and colleagues who used an RCAS retrovirus-mediated delivery [45]. Furthermore, expression of a truncated signaling-defective version of Serrate-1 also resulted in enhanced endocrine development, arguing that the effects we observe with full-length Delta1 and Serrate-1 is the result of a dominant negative effect. This was further corroborated by the ability of Notch1^ICD ^to reverse the effect of the ligands in a cell autonomous manner. However, in spite of the cell-autonomous effect on Notch signal reception, we would expect that cells lying adjacent to cells expressing Delta1 or Serrate1 would be prevented from differentiating through the non-cell autonomous activation of Notch signaling in neighboring cells, while this effect should not be observed for cells lying next to Serrate-d1-expressing cells which are signaling defective. However, since differentiating endocrine cells delaminate from the epithelium we were not able to ascertain whether differentiation of cells initially lying adjacent to Delta1- or Serrate1-expressing cells was delayed or inhibited.

It is noteworthy that we always observe that a fraction of the ligand expressing cells fail to undergo endocrine differentiation and that this fraction increases with time. One possible explanation for this is that these cells are differentiating into acinar cells as premature acinar differentiation is observed in zebrafish with compromised Notch signaling [19]; however, we found no evidence to suggest that amylase expression occurred prematurely. It is also possible that such cells are committed to a non-endocrine fate and that inhibition of Notch signaling therefore cannot induce endocrine differentiation. In this regard it is notable that forced expression of Ngn3 also fails to induce endocrine differentiation of all the cells that express the introduced gene. However, a trivial explanation may be that the cells that fail to differentiate express the introduced genes at levels that are incompatible with endocrine differentiation.

The endocrinogenic effect of Notch ligand expression was confined to the pancreatic endoderm. In spite of widespread expression in prospective duodenum we found no signs of endocrine development. This is most likely due to the restriction of *Ngn3 *expression to the pancreas anlage at this stage, but apparently contrasts with recent data from Gu and colleagues who found that forced expression of Manic Fringe in prospective duodenum led to endocrine development through inhibition of Notch signaling [46]. The reason for this discrepancy is unclear at present but could be related to the different mechanisms by which Manic Fringe and high levels of Delta1 inhibits Notch signal reception.

### Activated Notch inhibits endocrine promoting Ngn3 activity

Previous studies of Notch function in pancreatic endocrine development have not considered a potential role for Notch mediated inhibition of Ngn3 and/or NeuroD protein function. Several examples of post-translational inhibition of pro-neural function by Notch have been described in the literature. The function of the exocrine bHLH factor p48-PTF1 has been shown to be inhibited via heterodimerization to Hes1 [19]. In *Drosophila*, several Notch target genes of the E(Spl) class have been shown to dimerize with pro-neural bHLH factors and antagonize the function of these [14], in *Xenopus *X-NGN1 function can be antagonized by activated Notch1 [39,40], and in human cell lines Notch signaling has been demonstrated to lead to a rapid degradation of the human achaete-scute homolog hASH1 [15]. It is possible that similar mechanisms regulate Ngn3 function. It is also possible that regulatory sequences of genes induced by Ngn3 also contain Hes binding sites and that Hes repressor activity is dominant over Ngn3. Although our results do not prove that Notch signaling is active in Ngn3-expressing precursors they do raise the possibility that Notch might have a second effect in endocrine differentiation in that it could regulate the levels and duration of Ngn3 activity. A similar but not identical experiment has been conducted by Murtaugh and colleagues who used *Ngn3-*Cre mice to conditionally mix-express Notch1^ICD ^in endocrine progenitor cells which led to inhibition of endocrine differentiation [18]. However, since Ngn3 expression was not investigated in that study it is unclear whether this block in endocrine development was caused by inhibition of *Ngn3 *gene expression or Ngn3 protein activity. Recently, Notch1^ICD ^was shown to re-direct endocrine progenitors to a duct cell fate when expression was induced in *Pax4*-expressing cells [47]. Presumably, this effect would require inhibition of Ngn3 as *Pax4 *is a target of Ngn3.

A surprising finding is that activated Notch did not result in significant upregulation of *Hes1 *in the pancreas but rather appeared to repress the expression of this Notch effector gene. As discussed above this may be a secondary effect to the induction of *Hes5-1*. In a transgenic mouse expressing Notch1^ICD ^under the Pdx1 promoter, Hes1 protein could not be detected in most of the Notch1^ICD ^expressing pancreatic cells even though Ngn3 expression was almost completely abolished throughout the epithelium[17]. It was speculated that the Hes1 protein is expressed at levels too low for detection by immunohistochemistry. An alternative interpretation is that the effect of Notch1^ICD ^is mediated by other Hes factors such as Hes5, or that Notch signaling may be required but not sufficient for Hes1 expression under normal circumstances.

Surprisingly, Ngn3-induced delamination still occurred in the presence of Notch1^ICD^. The molecular mechanism controlling delamination of endocrine cells is poorly defined but it is possible that Ngn3-targets responsible for delamination are less sensitive to Notch-mediated repression than targets genes involved in endocrine differentiation, e.g., if genes required for delamination lack Hes binding sites in their regulatory elements. Intriguingly, differentiation and delamination is also uncoupled in other circumstances. A mutant Ngn3 protein carrying a missense mutation (R107S) is hypomorphic in regard to endocrine-inducing activity, but still capable of inducing delamination to the same extent as wild-type Ngn3 [48].

### Activated Notch antagonize Ngn3 as well as NeuroD function

Conditional expression of Notch1^ICD ^in mature insulin-producing beta cells has no apparent effect on the beta cell phenotype [18]. Since NeuroD function is required for beta cell survival and function [49,50] we speculated that NeuroD might be refractive to the effects of activated Notch. However, endocrine differentiation induced by forced NeuroD expression in chicken endoderm was completely prevented by co-expression of Notch1^ICD^. This could reflect a mechanistic change in NeuroD function in mature endocrine cells compared to precursor cells. In agreement with this notion we found that a few endocrine cells in the pancreas expressed Notch1^ICD ^probably reflecting the occasional transfection of existing endocrine cells at the time of electroporation. Alternatively, it is possible that the ROSA26 locus, used to conditionally express Notch1^ICD ^by Murtaugh and colleagues, results in appreciably lower levels of expression than we obtain by electroporation and that high level expression of Notch1^ICD ^is required for repression of NeuroD function. NeuroD is also capable of inducing neurogenesis when expressed in *Xenopus *ectoderm [39,51] but it is unclear if activated Notch can inhibit this function of NeuroD. In one study, X-NGNR-1 and XNeuroD were equally sensitive to inhibition by X-Notch^ICD ^in side-by-side comparisons [39], while in another study the number of neurons formed in response to X-NGNR-1 was reduced by X-Notch^ICD^which was not the case for XNeuroD induced neurons [40]. A number of observations support a similar function in pancreatic endocrine development; forced expression of either Ngn3 or NeuroD in the pancreas can induce the endocrine differentiation program [8,10,11] and Ngn3 induces *NeuroD *expression [10,11], while NeuroD cannot induce *Ngn3 *expression (this study) suggesting that a unidirectional bHLH cascade may also regulate endocrine determination and differentiation.

### MyT1 antagonism of Notch1^ICD ^activity

During *Xenopus *neurogenesis MyT1 can in cooperation with X-NGNR-1 overcome the repressive activity of Notch upon X-NGNR-1 [43]. We observe only a partial antagonistic effect of MyT1 on Notch mediated repression of Ngn3 function. The reasons for this incomplete rescue is unclear but it is possible that the chicken electroporation system results in far higher levels of activated Notch than seen with mRNA injection in Xenopus oocytes. It is possible that MyT1 would have a greater effect if more physiological expression levels were used. Nevertheless, the expression of known Notch signaling modifiers such as Hes6-1, Myt1, and Manic Fringe in endocrine precursors [44,46] suggests that Notch signaling is finely regulated during the early stages of endocrine differentiation. However, conditional loss-of-function studies in mice are needed to further elucidate the cellular and biochemical mechanisms of Notch signaling during these early stages of endocrine development.

## Conclusion

Together the data presented here establish that pancreatic endocrine development in the chicken relies in a conserved bHLH cascade under inhibitory control of Notch signaling. While this may not be surprising it does lay the ground for further studies that take advantage of the ease at which chicken embryos can be manipulated.

Our results also raise the possibility that Notch could act in Ngn3-expressing cells and delay their differentiation. To determine whether Notch in fact does act upon Ngn3 in vivo will require sophisticated conditional mutagenesis.

Lastly, our results demonstrate that expression of differentiation markers can be uncoupled from the process of delamination of differentiating cells from the epithelium.

## Methods

### DNA constructs

All expression constructs were made in a pCIG vector [52] optimized for EGFP intensity (pCIG5 or pCGIG5 with a Gateway (invitrogen) recombination cassette, kindly provided by Anne Grapin-Botton). A pcDNAI vector containing a cDNA encoding N-terminal haemaglutinin (HA) tagged mouse *Ngn3 *was kindly provided by François Guillemot. This vector was cut with HindIII and filled with klenow. The cDNA was released with NotI and the entire fragment was cloned into the EcoRV/NotI sites of pCIG5. The cDNA encoding N-terminally HA tagged rat *NeuroD *has been described previously [53]. The coding sequence was amplified by PCR with the primers CACCATGGGCTACCCATAGAT and CTAATCGTGAAAGATGGCATT and TOPO cloned into the pENTR/D vector (Invitrogen). Subsequently, HA-*NeuroD *was cloned by recombination into pCGIG5. The cDNA encoding C-terminally myc tagged rat Notch1^ICD ^has been described previously [17]. An EcoRI fragment containing the entire coding sequence was cloned into the EcoRI site of pCIG5. The mouse *Myt1*-7zf gene was FLAG tagged (MDYKDDDDKMTKSYSESGL) at the N terminus using PCR and cloned into pENTR/D (invitrogen). The tagged version of *Myt1*-7zf was cloned into the pCGIG5 vector using gateway cloning. The cDNAs encoding FLAG tagged chicken Delta1 and HA tagged chicken Serrate1 and the dominant negative Serrate1-d1 were a kind gift from Ken-ichi Katsube [28]. Delta1-FLAG was released with ApaI (Klenow blunted) and BamHI and ligated to EcoRI (Klenow blunted) and BamHI sites in pEntr 2B (Invitrogen). The coding sequence was cloned into the pCGIG vector by Gateway recombination. The Serrate constructs were cloned into XhoI and EcoRI sites of pEntr 2B and subsequently inserted in the pCGIG vector by Gateway cloning. All constructs were verified by sequencing.

### In ovo endoderm electroporation

Electroporations were performed as described in [11]. Briefly, fertilized White Leghorn eggs (Triova, Denmark) were incubated at 38°C, 50% relative humidity. Electroporation was performed on chicken embryos at HH st. 10–15 [23] : The eggs were windowed and a solution of DNA (with a fixed concentration of 2 μg/μl in PBS w/o Ca2+ and Mg2+, 1 mM MgCl2, 3 mg/ml carboxymethylcellulose, 0.66 mg/ml Fast Green) was injected into the blastocoel. An anode was placed over and a cathode under the embryo along the anteroposterior axis of the embryo using a micromanipulator. Depending on stage, three to five square 50 ms pulses of 7–15 V were applied from a BTX ECM830 electroporator and the eggs were sealed and returned to the incubator and allowed to develop for 52–72 hours. Electroporated embryos were immunostained with an antibody specific for the epitope tags and co-localization with GFP was assayed: As expected we found that most GFP positive cells also expressed the epitope tags (not shown) and we therefore used GFP as a measure of productive expression. For co-expression experiments double immunohistochemistry against the different epitope tags was used to verify co-expression (not shown).

### Immunohistochemistry and statistical analyses

Whole mount immunofluorescent stainings: The stomach with the proximal duodenum along with the pancreas was dissected from the embryos and fixed over night in 4% PFA at 4°C. Specimens were processed as in [54]. For IHC on sections, embryos were fixed in 4% PFA at 4°C over night, equilibrated in 30% sucrose in PBS and frozen in Tissue-Tek OCT compound (Sakura finetek). 10 μm sections were obtained on a Leica CM3050 S cryostat and collected on SuperFrost plus (Menzel-Gläser) slides. Sections were washed in PBS and blocked for at least 1 hour in 0.5% TNB (PerkinElmer). Primary antibodies were diluted in 0.5% TNB and applied over night. Secondary antibodies were applied for 1 hour after several washes in PBS and slides were mounted in 20% glycerol (in 50 mM Tris buffer adjusted to pH8.4). Primary antibodies were: Mouse-anti-HA (HA.11, Babco), rabbbit-anti-myc (A14, Santa Cruz), mouse-anti-FLAG (M2, Sigma), Mouse-anti-glucagon (Glu-001, Novo Nordisk A/S), guinea pig-anti-insulin (ab7842, Abcam), rabbit-anti-somatostatin (A566, DAKO), rabbit-anti-amylase (A 8273, Sigma), rabbit-anti-Laminin (L9393, Sigma), mouse-anti-MPM2 (M3514, DAKO), rabbit-anti-βIII-Tubulin (PRB-435P, Nordic Biosite). rabbit-anti-Nkx6.1 [55], rabbit-anti-GFP (632460, Clontech), (DSHB) The mouse anti-Pax6 monoclonal antibody developed by Atsushi Kawakami was obtained from the Developmental Studies Hybridoma Bank developed under the auspices of the NICHD and maintained by The University of Iowa, Department of Biological Sciences, Iowa City, IA 52242. Secondary antibodies were purchased from Jackson ImmunoResearch (Cy3 conjugated donkey-anti-rabbit and donkey-anti-guinea pig and Cy5 conjugated donkey-anti-mouse). Negative controls include omission of primary antibodies and staining of non-electroporated embryos Images were collected by confocal microscopy on an LSM 510 META Laser Scanning Microscope (Carl Zeiss) and fluorescent signals were assigned false colors with Zeiss LSM software. Quantification was performed by sampling sections covering all the electroporated endoderm (between 7 and 10 sections from each embryo) and counting at least between 350 and 750 GFP^+ ^cells from embryos electroporated at HH st. 10–12 and between 1200 and 4800 for embryos electroporated at HH st. 13–15. Between 3 and 13 embryos were analyzed quantitatively for each condition. Statistical analyses were performed using Student's T-test.

### In situ hybridization

Digoxigenin labelled cRNA probes were generated by in vitro transcription using reagents from Roche according to manufacturer's instruction. The following chicken cDNA clones were used for probe generation: *Ngn3 *was kindly provided by A. Grapin-Botton and J. M. Matter. *Delta1*, *Notch1*, *Serrate1 *and *Serrate2 *were generous gifts from D. Henrique [56,57]. *NeuroD*: ChEST695k11, *Hes1*: ChEST900h16, *Hes6-1*: ChEST296l7, *Hes6-2*: ChEST529e6, *Hes5-1*: ChEST295o19, *Myt1*: ChEST672b12, *Notch2*: ChEST909l22. ChEST clones were from the BBSRC Chick EST database and purchased from MRC gene service. Wholemount ISH was performed essentially as in [26]. ISH on frozen sections: Frozen sections were thawed and denatured cRNA probes diluted to 1 ng/μl in hybridization solution (50% formamide, 1x salt, 10% Dextran sulphate, 1 mg/ml yeast tRNA, 1x Denhardts) were added directly to the sections. The sections were covered with a cover slip and placed in a humidified chamber (humidified with 5×SCC, 50% formamide) at 65°C over night. The slides were washed 3 times in wash solution (50% formamide, 1× SCC, 0.1% Tween-20) at 65°C followed by several washes in MABT (100 mM maleic acid, 150 mM NaCl, 0.1% Tween-20, adjusted to pH7.5 with NaOH) at room temperature. The sections were blocked in 2% blocking reagent (Roche), 20% heat inactivated goat serum in MABT for 1 hour and incubated over night with an AP conjugated sheep-anti-DIG (Roche) (1:2500 in blocking solution). The sections were washed in MABT and then equilibrated in NTMT (50 mM Tris, 0.1 M NaCl, 0.02 M MgCl_2_, 0.1% Tween-20, pH9.5). AP activity was visualized with NBT/BCIP in NTMT. Images were collected with a Hamamatsu C5810 cooled CCD camera mounted on an Olympus BX51 microscope and processed in Adobe Photoshop™.

## Authors' contributions

JA-R designed and carried out most of the experiments. JH and AB performed the Notch ligand mis-expression. HY and JH-S cloned MyT1 and contributed to the mis-expression studies. JA-R and PS conceived the study, participated in its design and coordination, carried out the data analysis, and drafted the manuscript.
